# Osteoporosis screening and major osteoporotic fracture prediction by cranial computed tomography-derived Hounsfield units: a multi-center study on opportunistic osteoporosis screening

**DOI:** 10.1080/07853890.2025.2554930

**Published:** 2025-09-05

**Authors:** Robert Wakolbinger-Habel, Martin Bittner-Frank, Simon Desch, Clemens Lang, Brigitte Elisabeth Scheffold, Marc Reinmuth, Miroslava Cernakova, Robert Breuer, Manuela Schartel, Siroos Mirzaei, Klaus Hohenstein, Jakob Jauker, Daniel Buda, Karin Nagel-Albustin, Georg Berger, Nikolett Kainz, Max van Melle, Friedrich Lomoschitz, Helena Zehetner-Nics, Ana Oljaca, Theresa Zehetbauer, Barbara Strasser-Kirchweger, Tatjana Paternostro-Sluga, Mehdi Mousavi, Helmut Ringl, Daniel Arian Kraus, Rainer Fiala

**Affiliations:** a Department of Physical and Rehabilitation Medicine (PRM), Vienna Healthcare Group, Clinic Donaustadt, Vienna, Austria; b Department of Orthopaedics and Traumatology, Vienna Healthcare Group, Clinic Donaustadt, Vienna, Austria; c Department of Nuclear Medicine Diagnostics and Therapy, Vienna Healthcare Group, Clinic Donaustadt, Vienna, Austria; d Department of Nuclear Medicine with PET-Center, Vienna Healthcare Group, Clinic Ottakring, Vienna, Austria; e Department of Physical and Rehabilitation Medicine (PRM), Vienna Healthcare Group, Clinic Ottakring, Vienna, Austria; f Department of Physical and Rehabilitation Medicine (PRM), Vienna Healthcare Group, Clinic Hietzing, Vienna, Austria; g Department of Diagnostic and Interventional Radiology, Vienna Healthcare Group, Clinic Hietzing, Vienna, Austria; h Department of Physical and Rehabilitation Medicine (PRM), Vienna Healthcare Group, Clinic Floridsdorf, Vienna, Austria; i Department of Psychology, Faculty of Natural and Life Sciences, Paris Lodron University Salzburg, Salzburg, Austria; j Department of Diagnostic and Interventional Radiology, Vienna Healthcare Group, Clinic Donaustadt, Vienna, Austria; k Division of Endocrinology and Diabetology, Department of Internal Medicine, Medical University of Graz, Graz, Austria

**Keywords:** Cranial computed tomography, osteoporosis, dual X-ray absorptiometry, major osteoporotic fracture prediction, opportunistic screening

## Abstract

**Background:**

The incidence of osteoporosis and osteoporotic fragility fractures is increasing due to demographic changes. Therefore, early diagnosis is desirable in order to preserve bone health and prevent low-trauma fractures. Opportunistic screening for osteoporosis by frequently performed computed tomography scans could offer a potential solution. Cranial computed tomography is often performed in potential high-risk patients for osteoporosis due to frequent falls in older ages. The aim of this study was to examine the potential value of opportunistic osteoporosis screening by cranial computed tomography-derived Hounsfield units in the overall clinical context, including sustained major osteoporotic fractures, pre-existing diseases and lifestyle factors.

**Materials and methods:**

This retrospective multi-center study was conducted at the Vienna Healthcare Group, an association of the main clinics of the City of Vienna, Austria. The records of patients who received both a cranial computed tomography and a dual energy X-ray absorptiometry within one year were reviewed. The cranial computed tomography scans were assessed by two observers, and the mean Hounsfield Units were calculated for the frontal bone.

**Results:**

In total, 311 patients were analyzed. Hounsfield units significantly correlated with the T scores at the lumbar spine, total hip and femoral neck, and significant differences in Hounsfield units between patients with normal bone mineral density, osteopenia and osteoporosis were observed. These relationships remained significant in the geriatric subgroup. For the lumbar spine, 606 Hounsfield units were calculated as optimal cutoff value for the prediction of osteoporosis. Patients with sustained major osteoporotic fractures had significantly lower Hounsfield units (650) compared to patients without history of major osteoporotic fracture (715).

**Conclusions:**

Cranial computed tomography-derived Hounsfield units may assist to detect patients with densitometric osteoporosis and with major osteoporotic fractures at earlier stages, thereby facilitating early treatment and future fracture prevention.

## Introduction

Due to the aging society in industrialized countries, the incidence of osteoporotic fragility fractures is increasing. This not only leads to decreased quality of life in affected individuals, but also represents a high economic burden for the Austrian National Healthcare System [1]. Therefore, early diagnosis is desirable to preserve bone health and prevent low-trauma fractures.

However, as primary prevention of osteoporotic fractures is not approached sufficiently, many patients with osteoporosis receive osteoporosis treatment at the earliest after one or more sustained fragility fractures. Furthermore, as osteoporosis diagnosis is primarily based on bone density measurement by dual-energy X-ray absorptiometry (DXA), quick screening is hampered by the availability of these devices. Even though radiology services may be rather easily accessible for younger persons, the additional examination as well as longer travel distances are great burdens for the elderly. Moreover, additional radiologic examinations imply additional radiation exposure [2].

Therefore, opportunistic osteoporosis screening *via* routine radiological examinations obtained for other indications may provide additional benefits. Several studies investigated the potential value of computed tomography (CT)-derived Hounsfield Units (HU) in estimating DXA-based osteoporosis. Good to excellent correlations of HU of both the proximal femur and lumbar spine with DXA-derived BMD were reported. Furthermore, cutoff values for high-risk patients were established [2–9].

One of the most common CT-examinations performed at the clinics of the Vienna Healthcare Group is the cranial CT (CCT). This applies particularly to the geriatric population, mainly to rule out intracranial bleeding after falls, or in case of suspected stroke. Therefore, cranial CT scans could offer a particularly high benefit in opportunistic osteoporosis screening within the geriatric population to prevent low-trauma fractures, as the majority of hip, vertebral and radial fractures occur in persons older than 65 years [10].

In a Korean study, cranial CT derived HU were investigated. People aged 65 years and older had a lower mean HU value compared to younger individuals. Furthermore, a direct correlation of HU values and T scores was observed. In addition, a cutoff value (610 HU) for necessity of further evaluation concerning osteoporosis was established [11].

Another Korean study, focusing on hydrocephalus after subarachnoid hemorrhage, reported higher HU values in patients younger than 55 years compared to older ones [12].

In contrast, a Malaysian study evaluating multiple sites found CCT-derived HU to be no robust predictor for DXA-based BMD [4]. Furthermore, in a Croatian study on polytrauma patients, frontal bone HU did not vary with age [13].

A recent Australian study observed a moderate correlation of CCT-derived HU and DXA-based femoral neck BMD in women. Even though HU values did not vary with age and no optimal threshold was identified, HU <610 was highly specific for osteoporosis and HU >1200 in females had a strong negative predictive value [14].

None of these studies evaluated clinical parameters, such as sustained osteoporotic fractures, pre-existing diseases or lifestyle factors including alcohol and cigarette consumption; in addition, no serum markers of bone metabolism were analyzed.

Therefore, this study assesses the potential value of opportunistic osteoporosis screening by cranial CT derived HU in the overall clinical context.

## Methods

This retrospective multicenter study was conducted at six hospitals within the Vienna Healthcare Group, Austria.

Ethical approval was obtained from the local Ethics Committee (Ethikkommission der Stadt Wien, EK 24-030-VK), and the study was carried out in accordance with the Declaration of Helsinki (1964) and its subsequent amendments. Due to the retrospective character of the study, informed consent was waived by the local Ethics Committee.

Patients were identified through the Radiology Information System (Medavis, Siemens Healthineers, Erlangen, Germany). Included were patients who underwent both cranial CT and DXA examinations within one year, between 2014 and 2024. Patient records were reviewed accordingly. Most of included patients received CCT due to head trauma to rule out intracranial bleeding.

Cranial CT scans were acquired using various multi-detector CT scanners, including the Somatom Edge+, Somatom Definition AS, and Somatom Force (Siemens Healthineers). Scans followed a standardized protocol consistent with previously published methods [11]: 120 kV, 300–400 ref mAs with dose modulation, bone kernel (HR > 59), and a slice thickness of 2–3 mm.

**Figure 1.**
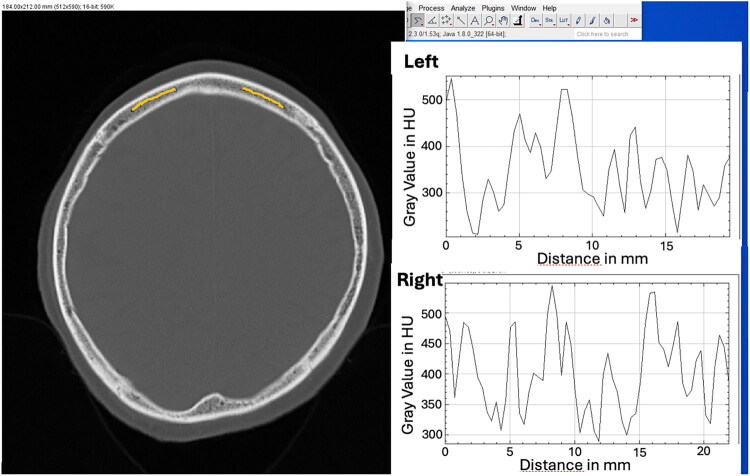
Linear histogram placement for HU measurement at the frontal bone.

CT images were transferred to the Syngo Plaza PACS (Siemens Healthineers) for analysis. Two independent readers assessed the images following established protocols [11], identifying a transverse skull section just above the lateral ventricles with adequate cancellous bone thickness (Figure 1). Therefore, only subjects with a sufficient thickness of the cancellous bone compartment were included. Both readers were blinded for both, the clinical data and the DXA results.

Selected sections were analyzed using ImageJ (University of Wisconsin; NIH). Each reader independently placed a curved line along the cancellous bone between the tabula externa and tabula interna of the frontal bone, carefully avoiding sutures and sinuses. The curved linear intensity profile in ImageJ was then utilized to calculate the mean Hounsfield Units (HU) across all points along the curve for each patient.

Demographic data, medical history, DXA results, and laboratory values were extracted from patients’ health records.

DXA scans were performed using either Prodigy (GE Healthcare, Chicago, IL, USA) or Horizon (Hologic Inc., Marlborough, MA, USA) systems. The T scores of the lumbar spine, femoral neck and total hip were obtained. The Trabecular Bone Score (TBS) was assessed using TBS iNsight^™^ (Medimaps Group, Plan-les-Ouates, Switzerland). Based on McCloskey et al. [15], TBS values were classified as follows: normal: >1.310; partially degraded: 1.230–1.310; degraded <1.230.

Exclusion criteria were frontal bone hyperostosis, history of skull fractures, skull surgery or intracranial foreign bodies, recent malignancy (within 5 years), severe renal insufficiency (CKD stages 4–5), genetic or metabolic bone disorders, and CT scans performed with contrast media.

### Statistics

The primary objective was to compare the mean HU values in patients of different age groups and to show possible correlations. Therefore, participants were divided into 5 age groups (50–59a, 60–69a, 70–79a, 80–89a and >90a) from which the statistical differences in HU values were determined. Descriptive statistics were represented by mean values and standard deviations. For determination of distribution, we graphically examined the data within each age group for normality using P–P plots and histograms.

The secondary objectives were to test for correlations of cranial CT derived HU values and DXA derived T scores., compare HU values of patients in the 3 densitometric classes (normal, osteopenia, osteoporosis), as well as in patients with and without sustained osteoporotic fractures, and test for correlations with the vitamin D status.

The DXA-derived T scores were categorized according to the WHO classification: T score ≤ −2.5 indicates osteoporosis, T score between −2.5 and −1 indicates osteopenia, and T score ≥ −1 indicating normal bone mineral density [16,17]. Linearity and normality requirements were checked using scatterplots, P–P plots, and histograms. All statistical analyses were performed using R (R Core Team, 2023).

The following statistical analyses were conducted:

First, interrater reliability was assessed using intraclass correlations (ICC), applying a two-way model focused on agreement for single measurements.

For the primary objective—the correlation and comparison between HU values and age—a Pearson correlation was performed. Additionally, differences in HU values across age groups were examined using ANOVA.

To explore the relationship between HU values and DXA derived T scores, Pearson correlations were calculated separately for each location (lumbar spine, femoral neck and total hip) as well as for the TBS score. Differences in HU values across DXA groups were further assessed *via* ANOVA, with post-hoc comparisons corrected for multiple testing using the Bonferroni method. These analyses were conducted both in the overall sample and in a subsample including only participants aged 70 years and older.

To investigate whether HU values differ among fracture types (no fracture, peripheral/vertebral fractures, femoral fractures), another ANOVA was performed.

Mean differences in HU values between smokers and non-smokers were analyzed using an independent samples Welch’s *t* test.

To assess the association between Vitamin D levels and HU values, a Spearman rank correlation was conducted. Additionally, group differences in HU values across Vitamin D categories were examined using ANOVA.

Finally, to determine whether HU values could serve as an indicator for osteoporosis, optimal cutoff values were identified by applying Youden’s Index to receiver operating characteristic (ROC) curves for each T score (lumbar spine, femoral neck, total hip) separately. The derived cutoff values were then applied to the data to evaluate the classification accuracy.

For all performed ANOVAs ω^2^ was calculated as effect size.

### Assessment of statistical power

Both a priori and post hoc power analyses were conducted to assess the statistical power of the study’s tests. The a priori power analysis indicated that the sample size was sufficient to detect significant effects for most planned statistical tests. Specifically, the a priori analysis estimated power values that were generally high, ensuring robust conclusions from the study. For example, the Pearson correlation (desired *n* = 67) and ANOVA (desired *n* = 100 per group) tests were planned with expected power values around 0.80, which is typically considered sufficient to detect meaningful effects.

The post hoc power analysis revealed that most of the conducted analyses were adequately powered. Specifically, correlations and ANOVA tests for T score and HU values (lumbar spine, femoral neck, total hip) demonstrated very high power (0.99), confirming strong relationships and group differences. However, the analysis of TBS and fracture types showed lower power (0.31 to 0.60), suggesting limited sensitivity to detect these effects. The power for the ANOVA comparing vitamin D levels and HU values was also modest (0.41), indicating that this test had limited ability to detect significant differences.

Overall, while some tests had lower power, particularly those involving TBS and fracture types, the majority of analyses were adequately powered, supporting the reliability of the study’s conclusions. Future studies with larger sample sizes or different methodologies may improve sensitivity, particularly for weaker effects. The post hoc power calculations are provided in supplementary document 1.

## Results

Of 498 patients, who met the inclusion criteria, 187 had to be excluded due to radiologic and/or clinical exclusion criteria. Therefore, 311 patients were included in the analysis. Mean time between CT scans and DXA scans was 115 ± 104 days.

The mean age of the included subjects was 73 ± 11 years. The predominant sex was female (67% female, 33% male). A quarter of the patients were diabetic, and less than every tenth insulin dependent. Current smoking was reported in one third of the patients. 70% of the patients had sustained at least one major osteoporotic fracture, but only 16% were on osteoporosis medication. Mean vitamin D status showed no severe insufficiency (26.3 ± 13.9 ng/ml). Vitamin D levels <20ng/ml were observed in 91 patients (34%), 20–30ng/ml in 65 patients (25%), and >30ng/ml in 108 patients (41%). Regarding the assessment of CT scans, the interclass correlation coefficient indicated good to excellent inter-rater reliability. The entire descriptive demographic data is displayed in Table 1.

**Table 1.** Descriptive basic data (*n* = 311), mean ± std or absolute and relative numbers.

Age (years)	73 ± 11
Females/males	207 (67%)/104 (33%)
Sustained major osteoporotic fractures	219 (70%)
Inflammatory diseases	138 (44%)
Diabetes mellitus	81 (26%)
Insulin-dependent diabetes mellitus	24 (8%)
Alcoholism	52 (17%)
Cigarette smoking	100 (33%)
Glucocorticoids >5mg prednisolone equivalent >3mo within last 2 years	49 (16%)
Antiresorptive or anabolic medication	51 (16%)
Vitamin D (ng/ml) (*n* = 264)	26.3 ± 13.9
Crosslaps (ng/L) (*n* = 141)	611 ± 380
Osteocalcin (ng/ml) (*n* = 107)	17.5 ± 9.5
Alkaline phosphatase (U/L) (*n* = 301)	100 ± 49
Procollagen 1 N-terminal propeptide (mcg/L) (*n* = 91)	91 ± 103
CT inter-rater reliability (interclass correlation coefficient)	0.80–0.82

**Figure 2.**
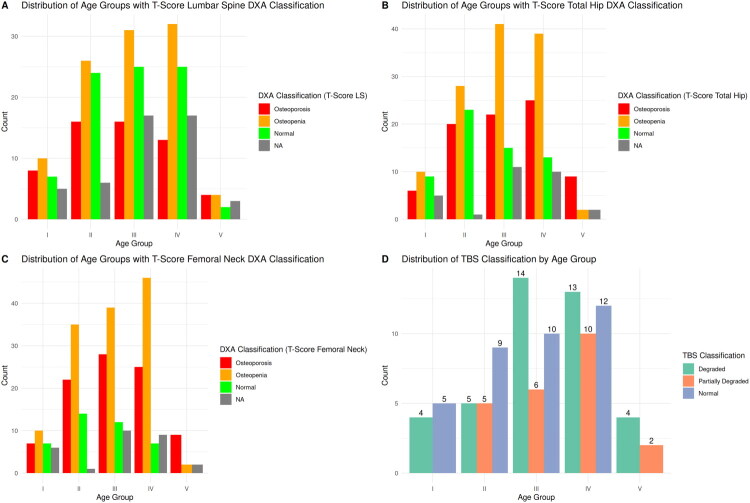
Distribution of bone mineral density and trabecular bone Score among the different age groups. Age groups were defined as: group 1 = 50–59 years, group 2 = 60–69 years, group 3 = 70–79 years, group 4 = 80–89 years, group 5 ≥ 90 years). Trabecular bone score grading: >1.31 = normal, 1.23–1.31 = partially degraded, <1.23 = degraded NA…. no exploitable results

Based on DXA derived BMD, most patients of the age groups I–IV (aged 50–89 years) belonged to the osteopenic range according to WHO. However, in age group V, the subjects were primarily osteoporotic. Entire DXA and TBS results are displayed in Figure 2.

**Figure 3.**
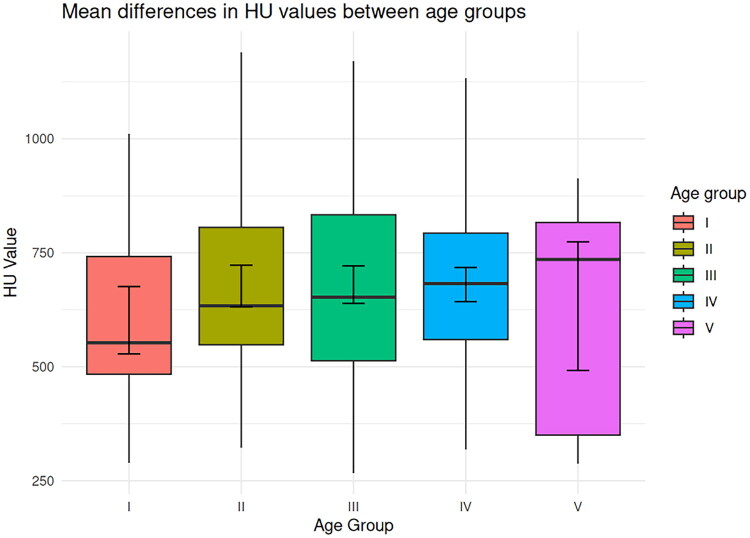
Mean differences in HU values between the different age groups (group 1 = 50–59 years, group 2 = 60–69 years, group 3 = 70–79 years, group 4 = 80–89 years, group 5 ≥90 years).

There was no significant correlation of HU with age (*r* = 0.088, *p* = 0.133). In a separate analysis of women and men, still no significant correlations were observed (*r* = 0.088, *p* = 0.133 for both genders). Similarly, the ANOVA showed no significant differences in HU values between the age groups (*F* = 1.211, ω^2^ = 0.003, *p* = 0.306). Figure 3 provides the mean differences in HU values between the different age groups as boxplots.

We observed significant correlations of HU values with DXA-derived T scores. However, the correlation coefficients were rather weak (lumbar spine *r* = 0.368, total hip *r* = 0.34, femoral neck *r* = 0.298). For the Trabecular Bone Score, no significant correlations were found (*p* = 0.142).

Furthermore, we observed significant HU-differences between patients with DXA-based normal BMD, osteopenia and osteoporosis at each measurement location.

In the geriatric subgroup of patients >70 years (*n* = 138, mean 79 years), the correlations of HU with DXA-derived T scores remained significant, as well as the differences between patients with DXA-based normal BMD, osteopenia and osteoporosis. In addition, a significant weak correlation with the trabecular bone score (TBS) was observed (*r* = 0.279).

**Figure 4.**
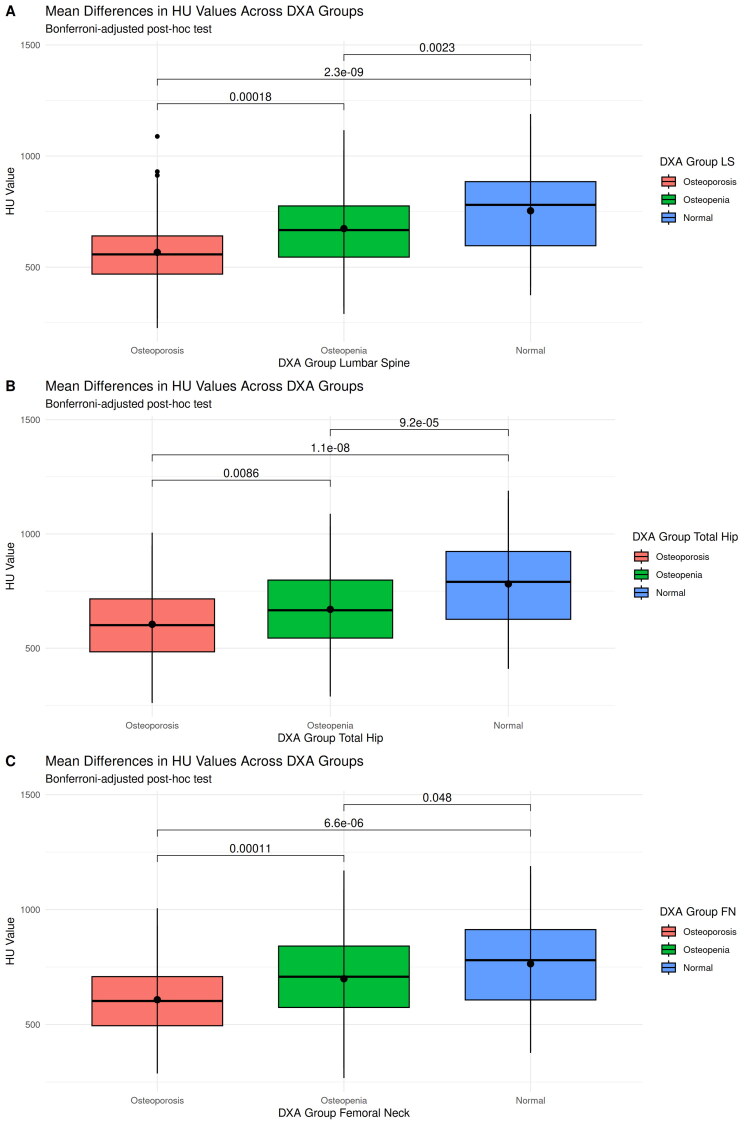
Bonferroni-adjusted post hoc tests of the relationships of HU with DXA-derived T score categories. Panel A: ANOVA post hoc tests for group comparison of HU means between lumbar spine T score categories. Panel B: ANOVA post hoc tests for group comparison of HU means between total hip T score categories. Panel C: ANOVA post hoc tests for group comparison of HU means between femoral neck T score categories.

The results are displayed in Table 2 and Figure 4, the correlation plots are provided in Supplementary Figure 1.

**Table 2.** Correlation analysis of HU and DXA-derived T scores, and ANOVA-results between patients with DXA-based normal BMD, osteopenia and osteoporosis. The upper part provides the results for the whole sample, the lower part for the geriatric subgroup of patients >70years.

	Correlation coefficient (*r*)	Correlation *p* value	ANOVA *F* value	ANOVA effect size (ω²)	ANOVA *p* value
Lumbar spine	0.368	<0.001	20.061	0.127	<0.001
Total hip	0.34	<0.001	18.649	0.112	<0.001
Femoral neck	0.298	<0.001	13.369	0.081	<0.001
Trabecular Bone Score (*n* = 106)	0.142	0.142	N/A	N/A	N/A
**Geriatric subgroup >70 years (***n* = **138)**					
Lumbar spine	0.364	<0.001	13.04	0.149	<0.001
Total hip	0.264	0.002	7.938	0.091	0.001
Femoral neck	0.232	0.006	4.047	0.042	0.02
Trabecular bone score (*n* = 61)	0.279	0.027	N/A	N/A	N/A

Table 3 provides optimal HU cutoff values for the estimation/classification of DXA-based osteoporosis.

**Table 3.** Optimal HU cutoff values for the estimation/classification of DXA-based osteoporosis at the lumbar spine, total hip and femoral neck.

	Optimal cutoff value	Sensitivity	Specificity
Lumbar spine	606	72%	69%
Total hip	667	71%	56%
Femoral neck	652	68%	57%

Optimal cutoff values for the total hip and femoral neck were 667 and 652 HU, respectively.

For the lumbar spine an optimal cutoff value of 606 HU was calculated, with a sensitivity of 72% and specificity of 69%. Based on these cutoff values, 69% of our patients with osteoporosis at the lumbar spine were classified correctly, 61% of our patients with osteoporosis at the total hip, and 60% of our patients with osteoporosis at the femoral neck. Supplementary Figure 2 provides ROC curves and cutoff graphs.

**Figure 5.**
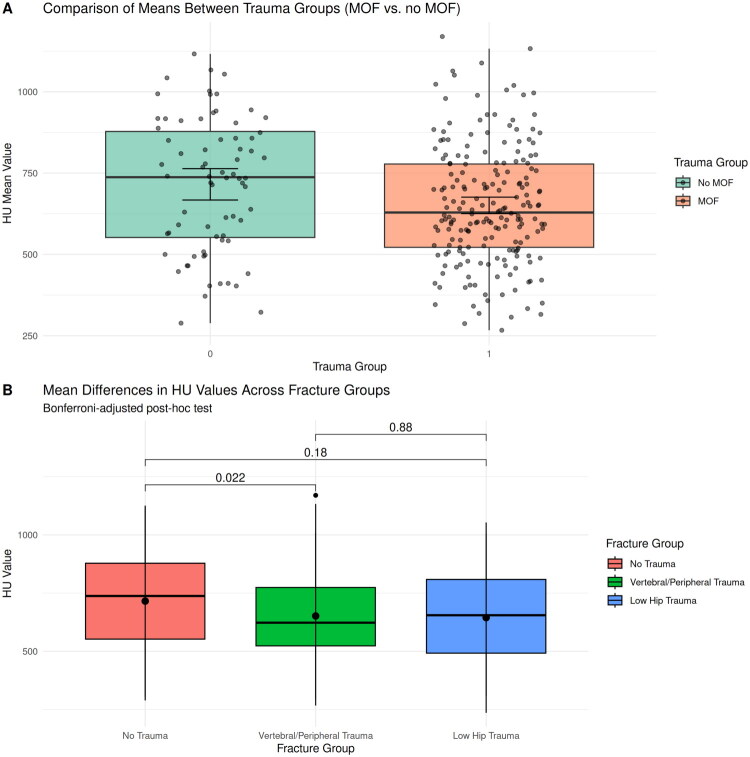
HU differences between patients with and without sustained major osteoporotic fracture (MOF). Panel A: Comparison of mean HU values between patients with and without MOF. Panel B: Subgroup analysis between patients without history of fracture, patients with low trauma vertebral or peripheral fracture, and patients with low trauma hip fracture. HU values were significantly lower in patients with sustained vertebral or peripheral fracture, compared to patients without history of fracture.

Patients with sustained major osteoporotic fractures (MOF) had significantly lower HU values (650) compared to patients without history of MOF (715; *p* = 0.019). The ANOVA confirmed the differences (*F* = 3.139, ω^2^ = 0.016, *p* = 0.045). In a subgroup analysis (group 1 = no fracture; group 2 = low trauma vertebral or peripheral fracture, group 3 = low trauma hip fracture), a significant difference in HU values between groups 1 and 2 were observed. Figure 5 demonstrates the differences of mean HU values as boxplots, as well as Bonferroni-adjusted post hoc test.

Furthermore, patients with current cigarette consumption had significantly lower HU values (628) compared to non-smokers (692, *p* = 0.004). No significant correlation of serum vitamin D levels and HU values were found (rho = −0.03, *p* = 0.63). For the ANOVA, vitamin D status groups were defined as deficiency (<20 ng/ml), insufficiency (20–30 ng/ml), and normal (>30 ng/ml). No significant HU-differences between the groups were observed (*p* = 0.137).

## Discussion

To the best of our knowledge, this study on opportunistic osteoporosis screening *via* CCT is the first one assessing not only radiological, but also clinical and laboratory data and thereby incorporating numerous relevant fracture-associated parameters. In addition, this is the first European multi-center study in this field.

As most important outcome, we observed a significant difference in HU values based on densitometric classification of normal, osteopenia and osteoporosis. In literature, there is an emphasis on vertebral BMD and HU, showing that DXA-defined osteoporotic subjects have significant lower HU values on native CT scans of the spine [18]. However, DXA is currently still seen as the gold standard for bone mineral density evaluation despite major limitations [19]. One major issue is the high fragility fracture rate of patients with osteopenic BMD. This discrepancy could potentially be overcome using HU as a marker for bone fragility. Colleagues showed that the HU are unaffected from degenerative changes of the lumbar spine [20], indicating the potential of HU based bone fragility evaluation compared to DXA scans. Recently it was shown, that osteoanabolic agents (Teriparatide, Romosozumab) taken for approximately the recommended period exposed an increase in CT based HU values of the spine [21], indicating also the power of HU based evaluation in follow up assessment of therapy success. Furthermore, patients with a previous major osteoporotic fracture (hip, vertebral, pelvic, radius, humerus) had significantly reduced HU compared to non-fractured patients, indicating the presence of manifest osteoporosis. As every MOF increases the risk of additional fractures, early identification is desirable to initiate proper antiresorptive or osteoanabolic treatment. Based on our data, opportunistic screening *via* CCT may be a useful tool for the early identification in primary prevention as well as secondary prevention, ideally incorporated in a systematic approach such as a fracture liaison service (FLS). In Austria, currently only 16% of therapy naïve women and 12% of therapy naïve men received a specific antiosteoporotic treatment 1 year after a MOF, emphasizing the need of systematic approaches [22].

Besides that, we also observed a significant correlation between HU and DXA-derived T scores at each measurement site (lumbar spine, femoral neck, total hip). Although these correlations were significant, the correlation coefficient only showed a weak correlation at each measurement site. This is also supported by other CCT studies in literature. Na et al. [11] reported a significant correlation of HU and DXA in the Korean population. Lim et al. [14] reported fair correlations in an Australian sample. In contrast, a Malaysian study [4] observed a low and marginally significant correlation. However, none of these studies included clinical parameters such as chronic conditions, and there were no clinical exclusion criteria. Moreover, the methods of CCT analysis differed. Similar to Na et al. we used a linear line intensity profile, whereas both other studies used a region of interest (ROI). Based on the difference in correlations, the line intensity profile may be more useful in this context.

For the Trabecular Bone Score (TBS), we observed no significant correlations in the entire cohort. In contrast, a significant correlation was found in the geriatric subgroup. To the best of our knowledge, there are no previous studies on CT-derived HU and TBS. Therefore, this potential relationship should be examined in a larger sample size.

Studies on CT-screening at the lumbar spine and hip reported higher correlation coefficients with DXA (0.49–0.76), compared to our study [4,5,7,9]. These findings are probably explained by the fact, that CT- and DXA-images from the same bones have been obtained and analyzed. In contrast, the development, structure and biology of the skull are distinct [14], which may explain the lower correlation coefficients in our study.

Due to significant correlations of CT-based HU and DXA, artificial intelligence and deep learning systems are investigated for automatic CT screening. Recent studies on spinal CT reported promising results [23–25]. Based on our data, we defined optimal HU cutoff values for the estimation/classification of DXA-based osteoporosis e.g. 606 HU at the lumbar spine with a sensitivity of 72% and specificity of 69%. Na and colleagues [11] reported a very similar optimal cutoff value (610 HU), while in the study by Amin and coworkers [4] it was lower (537 HU). Lim et al. [14] could not define an optimal cutoff value based on their results. These differences may be explained by the distinct methods of CCT analysis. However, with the cutoff values based on our results we suggest a reasonable threshold for osteoporosis screening.

In all previous studies on CCT, the patients’ mean age was <70 years. Our study is the first one specifically analyzing the potential value of the CCT in the geriatric population >70 years. As the geriatric population is vulnerable due to increased risk of falls and fractures, opportunistic screening is of particular importance for this group to prevent osteoporotic fractures. Due to frequent necessity for routine CCT, the potential benefit of opportunistic screening may be remarkable.

As age is one of the most important unmodifiable risk factors for osteoporosis [26], surprisingly our study showed neither a significant correlation of HU and age, nor a significant difference of HU values between different age groups.

A possible explanation could be the high prevalence of osteopenia in each age group, indicating a potential selection bias. Another explanation could be the low statistical power. For the correlation between age and HU values, the post-hoc power was found to be 0.32, indicating a low power for detecting a significant relationship. The ANOVA assessing the differences in HU values across age groups had a slightly higher power of 0.39, but still indicating a modest ability to detect group differences.

Furthermore, the skull has special characteristics concerning bone biology and development, compared to other skeletal sites, which may influence the relationship of HU with age [14]. However, also in other studies, no correlation with age was seen [12,13].

Other evaluated risk factors of osteoporosis include smoking, diabetes, corticosteroid intake, and vitamin D status.

We showed that smoking has a systemic effect on the skeleton through significantly decreased HU values compared to non-smokers. To our knowledge, there is only one previous study on the relationship between smoking and CT-derived HU, reporting a stronger decline in spinal HU in current than former smokers [27].

We observed no significant correlation of serum vitamin D levels with HU values. These findings may be explained by the fact that vitamin D deficiency increases fracture risk beyond BMD [28,29]. In particular, bone microarchitecture and quality are affected, which are both not fully reflected by DXA [30].

Due to the retrospective character of this study, certain limitations exist, such as missing information (e.g. some medications and diagnoses not listed in the discharge summaries, osteoporosis disease duration, as well as conditions and fractures treated outside the Vienna Healthcare Group). Moreover, the samples consisted mainly of women with densitometric osteopenia. In addition, retrospective evaluations are prone to selection bias, which could explain the missing correlation of HU with age in our sample. Furthermore, CCT and DXA were performed at different time points and the time intervals varied up to one year. However, as the maximal time interval was up to one year, we assume only a small distortion of the results. Besides the limitations, our study also has several strengths: All CCT-images were reviewed by two observers to ensure high analytic quality as well as reproducibility of results. Furthermore, we included numerous osteoporosis-associated clinical parameters including history of MOF and serum parameters.

Further studies, especially prospective studies with greater sample sizes are required for evaluation of daily routine applicability. Additionally, future studies should also focus on artificial intelligence-based techniques for opportunistic osteoporosis screening *via* CCT scans and linking them to systematic approaches for primary and secondary fracture prevention to improve patient care.

In conclusion, opportunistic osteoporosis screening based on CCT-derived HU potentially detects patients at risk of densitometric osteoporosis or with manifest osteoporosis at earlier stages, thereby paving the path for early treatment and future fracture prevention. Further study of bone density by DXA should be performed in those with a low HU value.

## Supplementary Material

Supplemental Material

Suppl figure 1.tiff

Supplementary document 1.docx

Suppl figure 2a.tif

Suppl figure 2b.tif

## Data Availability

Data supporting the findings of this study are available upon reasonable request.
