# Supplementary material for: Osteoporosis screening and major osteoporotic fracture prediction by cranial computed tomography-derived Hounsfield units: a multi-center study on opportunistic osteoporosis screening
Source: Ann Med. 2025 Sep 5;57(1):2554930. doi: 10.1080/07853890.2025.2554930 (PMC12416018; doi:10.1080/07853890.2025.2554930)
Supplement: Supplemental Material [file IANN_A_2554930_SM2172.docx]

Supplementary figure 1: Correlation plots of the relationships of HU with DXA-derived T-Scores. Panel A: Correlation plot for lumbar spine BMD. Panel B: Correlation plot for total hip BMD. Panel C: Correlation plot for femoral neck BMD.

Supplementary figure 2: ROC curves (A-C) and apply cutoff graphs (D-F) for the optimal HU cutoff values for the classification of DXA-based osteoporosis at the lumbar spine, femoral neck and total hip. Panel A: ROC curve for the lumbar spine. Panel B: ROC curve for the femoral neck. Panel C: ROC curve for the total hip. Panel D: Apply cutoff to predict osteoporosis at the lumbar spine. Panel E: Apply cutoff to predict osteoporosis at the femoral neck. Panel F: Apply cutoff to predict osteoporosis at the total hip.
