# Supplementary figures and images for: Osteoporosis screening and major osteoporotic fracture prediction by cranial computed tomography-derived Hounsfield units: a multi-center study on opportunistic osteoporosis screening
Source: Ann Med. 2025 Sep 5;57(1):2554930. doi: 10.1080/07853890.2025.2554930 (PMC12416018; doi:10.1080/07853890.2025.2554930)

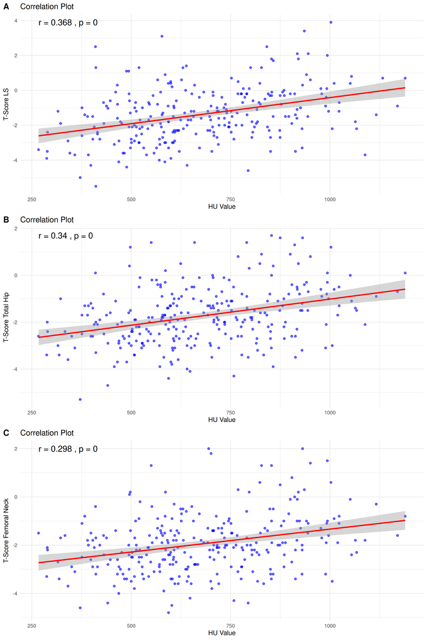

Supplement: Suppl figure 1.tiff [file IANN_A_2554930_SM1441.tiff]

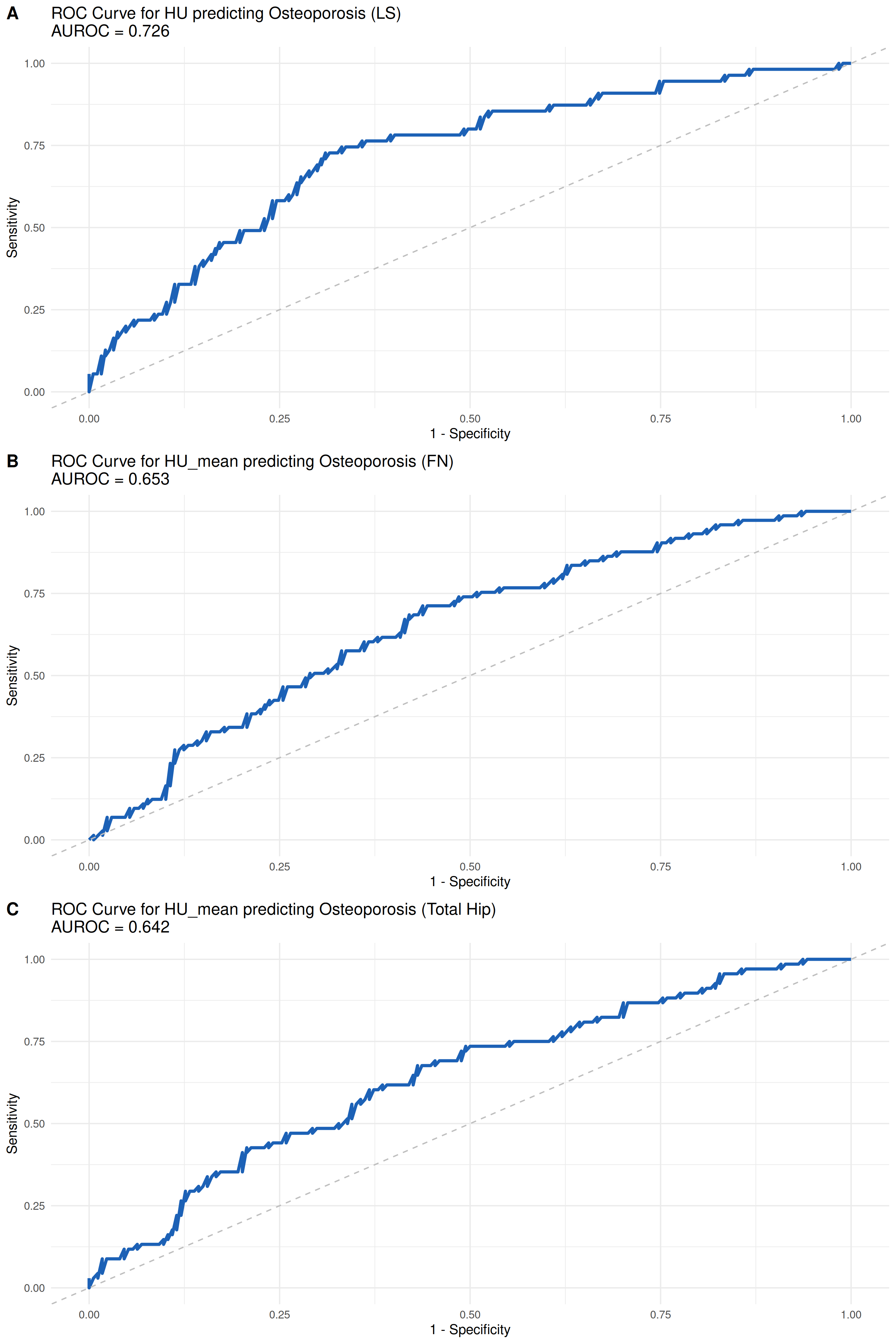

Supplement: Suppl figure 2a.tif [file IANN_A_2554930_SM1439.tif]

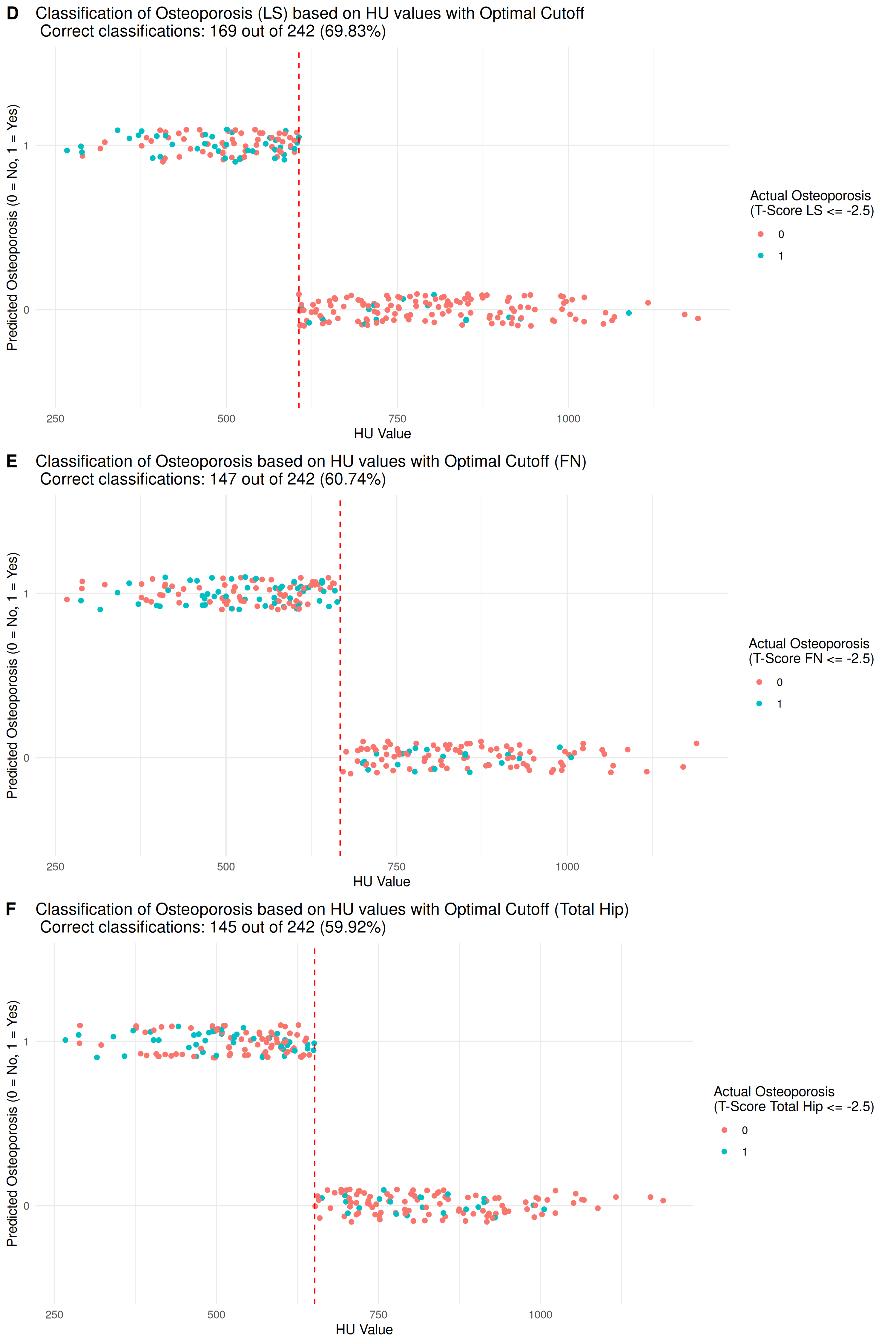

Supplement: Suppl figure 2b.tif [file IANN_A_2554930_SM1438.tif]
