# Supplementary material for: Osteoporosis screening and major osteoporotic fracture prediction by cranial computed tomography-derived Hounsfield units: a multi-center study on opportunistic osteoporosis screening
Source: Ann Med. 2025 Sep 5;57(1):2554930. doi: 10.1080/07853890.2025.2554930 (PMC12416018; doi:10.1080/07853890.2025.2554930)
Supplement: Supplementary document 1.docx [file IANN_A_2554930_SM1440.docx]

Supplementary document 1: Post-hoc power calculations

To assess the statistical power of the conducted analyses, post-hoc power calculations were performed for each hypothesis and statistical test used. These calculations provide an estimate of the likelihood that the tests would correctly detect an effect, assuming the effect exists. The results of the power analyses are summarized below.

Hypothesis 1: Age and HU Values

For the correlation between age and HU values, the post-hoc power was found to be 0.32, indicating a low power for detecting a significant relationship. The ANOVA assessing the differences in HU values across age groups had a slightly higher power of 0.39, still indicating a modest ability to detect group differences in this analysis.

Hypothesis 2: T-Score and HU Values (ANOVA across DXA Groups of T-Score)

The correlation between HU values and the T-Score for various body regions (lumbar spine, femoral neck, total hip) showed very high post-hoc power, with values exceeding 0.99 for all three regions. This suggests strong evidence for a relationship between T-Score and HU values. Similarly, the ANOVA tests assessing differences in HU values across DXA groups also displayed high power (0.99). However, the correlation for TBS was notably lower, with a power of 0.31, suggesting limited ability to detect a significant relationship between TBS and HU values.

Hypothesis 2.2: T-Score and HU Values (ANOVA across DXA Groups of T-Score for Participants Aged > 70)

When restricting the analysis to participants over the age of 70, the power for the correlation between HU values and the T-Score of the lumbar spine remained very high (power = 0.99), and the ANOVA for lumbar spine also showed similarly high power (0.99). However, the power for the correlation and ANOVA tests for the total hip and femoral neck were slightly lower, with values ranging from 0.72 to 0.96. The power for the correlation for TBS was again lower (0.60), indicating weaker evidence for a relationship between TBS and HU values in this age group.

Hypothesis 4: Comparison of Fracture Types

The power for the ANOVA test comparing HU values across different fracture types (no trauma, peripheral fractures, femur fractures) was 0.60, suggesting a moderate ability to detect group differences in HU values across fracture types.

Hypothesis 5: Smokers vs Non-Smokers in HU Values

For the comparison of smokers versus non-smokers, the independent samples t-test assessing differences in HU values had a post-hoc power of 0.82, indicating a high likelihood of detecting a significant difference between these groups.

Hypothesis 6: Vitamin D Levels and HU Values

The correlation between Vitamin D levels and HU values demonstrated a high power of 0.82, indicating a strong relationship between these variables. However, the ANOVA comparing HU values across Vitamin D groups had a lower power of 0.41, suggesting that the test had limited sensitivity for detecting differences between groups in HU values based on Vitamin D levels.

Age x HU:

Correlation: Power=0.32

ANOVA: Power=0.39

T-Score x HU values (ANOVA DXA groups of T-Score):

Correlation LWS: Power = 0.99

ANOVA LWS: Power = 0.99

Correlation Femur: Power = 0.99

ANOVA Femur: Power = 0.99

Correlation SH: Power = 0.99

ANOVA SH: Power = 0.99

Correlation TBS: Power = 0.31

T-Score x HU values (ANOVA DXA groups of T-Score) > 70 years:

Correlation LWS: Power = 0.99

ANOVA LWS: Power = 0.99

Correlation Femur: Power = 0.88

ANOVA Femur: Power = 0.96

Correlation SH: Power = 0.77

ANOVA SH: Power = 0.72

Correlation TBS: Power = 0.60

MOF x no history of MOF:

ANOVA: Power = 0.60

Smokers vs non-smokers in HU values:

T-Test: Power = 0.82

Vitamin D levels and HU values:

Correlation: Power=0.82

ANOVA: Power = 0.41
